# Supplementary material for: Two stages of substrate discrimination dictate selectivity in the Escherichia coli MetNI-Q ABC transporter system
Source: J Biol Chem. 2025 Jul 17;301(8):110493. doi: 10.1016/j.jbc.2025.110493 (PMC12357092; doi:10.1016/j.jbc.2025.110493)
Supplement: Supporting information [file mmc1.pdf]

Supporting information

Two stages of substrate discrimination dictate selectivity in the *E. coli* MetNI-Q ABC transporter system

Janet G. Yang, Hulda Yuchun Chen, John H. Guardado, Maile Gardner, and Matthew S. Foronda

Supporting figures 1-5

Figure S1

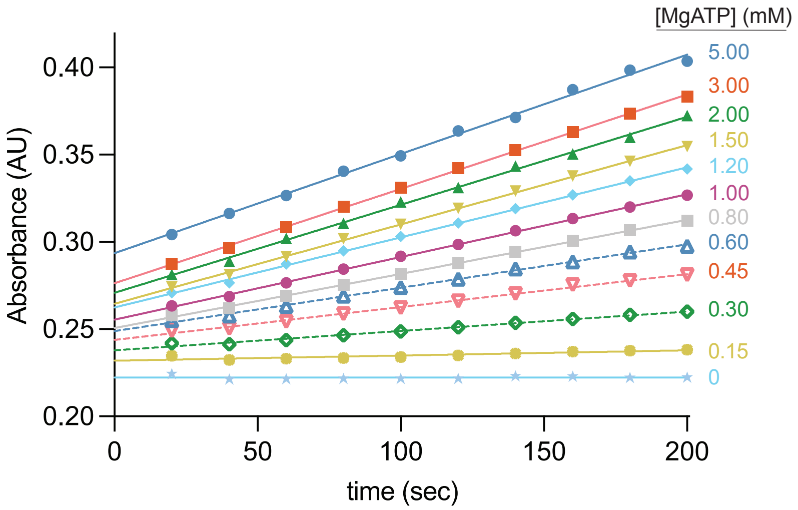

Supporting Figure 1. Representative raw data from ATPase assays. MgATP was mixed with detergent-solubilized MetNI and the absorbance at 360 nm was measured for 200 seconds. Reactions contained a final concentration of 500 nM MetNI and the indicated final concentration of MgATP.

Figure S2

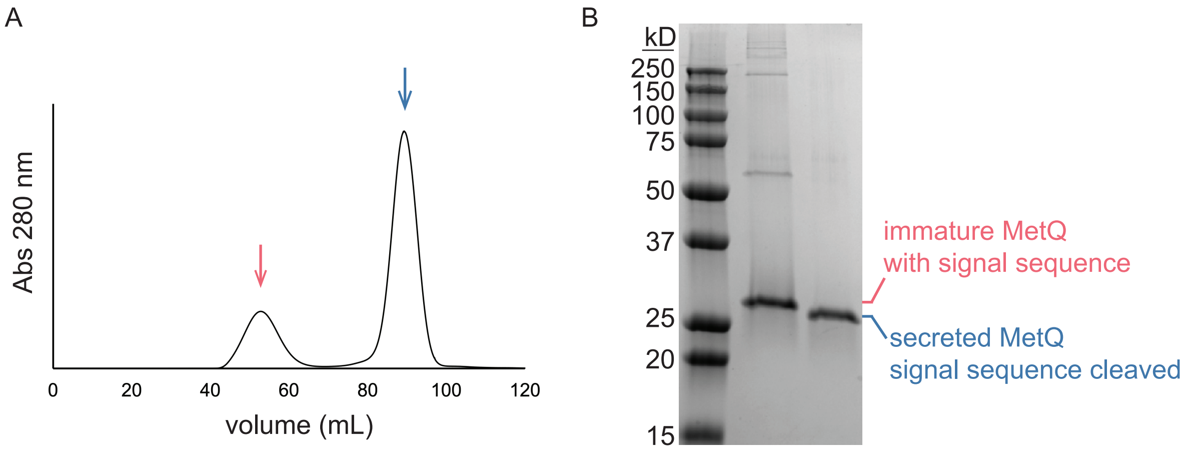

Supporting Figure 2. Purification of secreted MetQ. (a) Chromatogram of Ni-affinity purified His-MetQ injected onto a HiLoad 16/600 Superdex 200 pg column. The red arrow indicates insoluble material eluting at the void volume. The blue arrow highlights a monodisperse peak at ~90 ml elution volume. (b) SDS-PAGE of SEC fractions. Secreted MetQ was used in all experiments in this study.

Figure S3

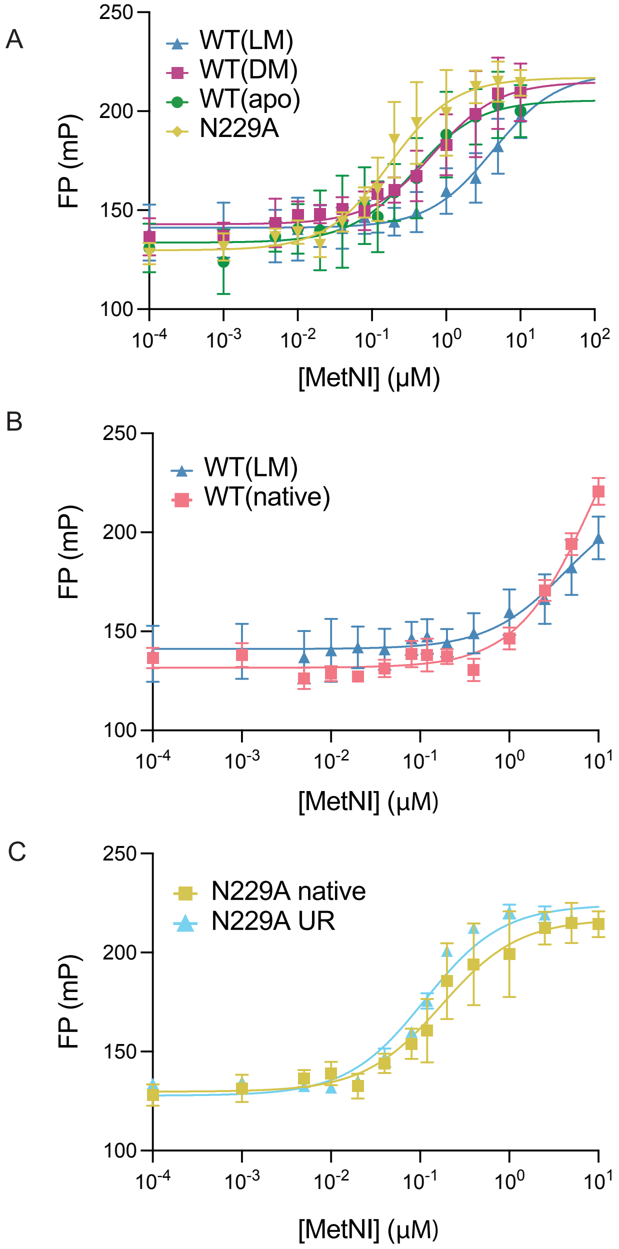

Supporting Figure 3. Raw data from anisotropy experiments. MetNI-Q complex formation as a function of substrate. Reactions contain 20 nM labeled MetQ**,** DDM-solubilized E166Q/N295A MetNI**,** and ATP/EDTA. (a) Raw data corresponding to Figure 4e in the main text. (b) Comparison of purified native WT (red), which copurifies with L-Met, and unfolded-refolded (UR) WT with 100 μM exogenous L-Met added (blue). K_d values are 8 ± 2 μM and 5 ± 3 μM, respectively. (c) Comparison of purified native N229A (yellow) and unfolded-refolded (UR) N229A (cyan). K_d values are 0.3 ± 0.2 μM and 0.113 ± 0.007 μM, respectively. Data were fit to a one-site specific binding model to determine the K_d and maximum and minimum FP values (see Materials and Methods).

Figure S4

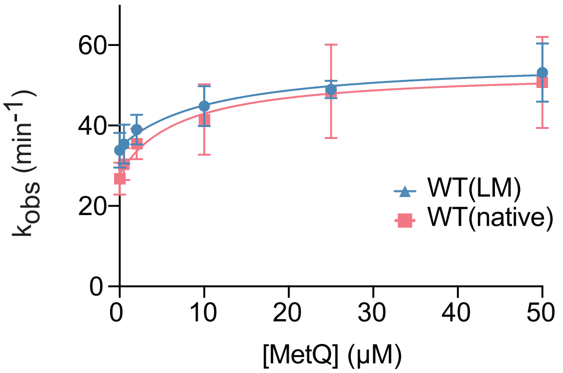

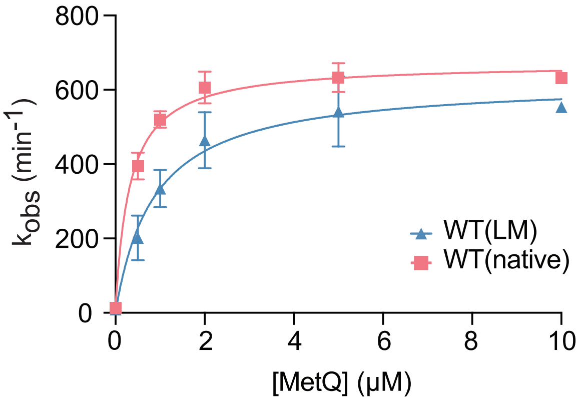

Supporting Figure 4. MetQ stimulated ATPase activity of MetNI. (a)100 nM detergent-solubilized N295A MetNI was incubated with 5 mM MgATP and 0 - 50 μM MetQ. Native WT MetQ containing co-purified L-Met (red) was compared with unfolded-refolded WT MetQ with 100 μM exogenous L-Met (blue). K_m(MetQ) values are 6 ± 2 μM and 14 ± 9 μM, respectively. k_{cat}(MetQ) values are 50 ± 20 min^{-1} and 60 ± 10 min^{-1}, respectively. (b) ATPase activity using 32 nM N295A MetNI nanodiscs, 5 mM MgATP, and 0 - 10 μM MetQ. Kinetic parameters of native WT MetQ (red) and unfolded-refolded WT MetQ with 100 μM exogenous L-Met (pink) are as follows: K_m(MetQ) values are 0.3 ± 0.1 μM and 0.9 ± 0.2 μM, respectively. k_{cat}(MetQ) values are 660 ± 30 min^{-1} and 630 ± 90 min^{-1}, respectively.

Figure S5

A

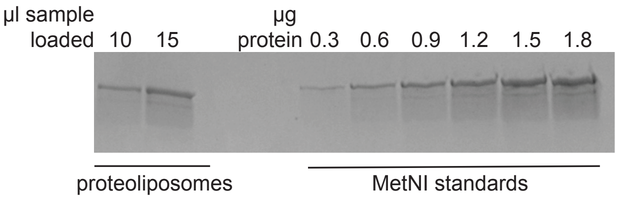

B

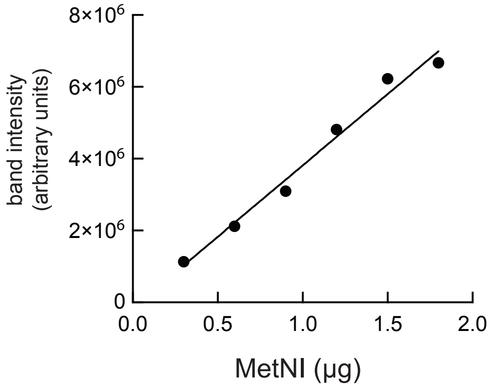

Supporting Figure 5. Representative SDS-PAGE for liposome quantification. (a) Coomassie-stained gel containing different amounts of a liposome preparation compared to known standards. (b) Standard curve for interpretation of data in (a). R^2 value is 0.9822.
